# 6′-Bromo-1′*H*-spiro­[cyclo­hexane-1,2′-pyrido[2,3-*d*]pyrimidin]-4′(3′*H*)-one

**DOI:** 10.1107/S1600536811052299

**Published:** 2011-12-21

**Authors:** Liupan Yang, Daxin Shi, Shu Chen, Hongxin Chai, Jiarong Li

**Affiliations:** a School of Chemical Engineering and Environment, Beijing Institue of Technology, Beijing 100081, People’s Republic of China

## Abstract

The title compound, C_12_H_14_BrN_3_O, is built up from two fused six-membered rings and one six-membered ring linked through a spiro C atom. The hydro­pyrimidine ring has an envelope conformation and the cyclo­hexane ring is in a chair conformation. In the crystal, mol­ecules are linked by N—H⋯O and N—H⋯N hydrogen bonds, forming a mol­ecular tape along the *b* axis.

## Related literature

For medicinal and biological properties of 2,3-dihydro­pyrido[2,3-*d*]-pyrimidin-4(1*H*)-one derivatives, see: Parish *et al.* (1982 ▶); Narayana *et al.* (2009 ▶). For related structures, see: Shi *et al.* (2010 ▶); Ling *et al.* (2009 ▶).
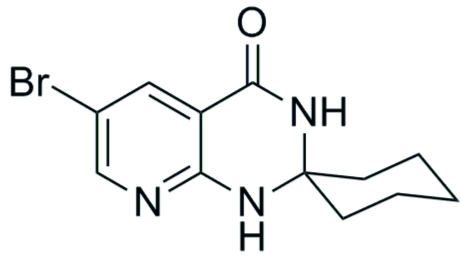


## Experimental

### 

#### Crystal data

C_12_H_14_BrN_3_O
*M* *_r_* = 296.17
Monoclinic, 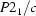

*a* = 10.591 (3) Å
*b* = 12.359 (3) Å
*c* = 9.116 (3) Å
β = 97.951 (4)°
*V* = 1181.7 (6) Å^3^
*Z* = 4
Mo *K*α radiation
μ = 3.47 mm^−1^
*T* = 153 K
0.40 × 0.24 × 0.09 mm

#### Data collection

Rigaku AFC10/Saturn724+ diffractometer
Absorption correction: multi-scan (*CrystalClear*; Rigaku/MSC, 2009 ▶) *T*_min_ = 0.324, *T*_max_ = 0.732
10128 measured reflections
3160 independent reflections
2283 reflections with *I* > 2σ(*I*)
*R*_int_ = 0.046

#### Refinement

*R*[*F* ^2^ > 2σ(*F* ^2^)] = 0.035
*wR*(*F* ^2^) = 0.074
*S* = 1.00
3160 reflections
162 parameters
H atoms treated by a mixture of independent and constrained refinement
Δρ_max_ = 0.55 e Å^−3^
Δρ_min_ = −0.66 e Å^−3^

### 

Data collection: *CrystalClear* (Rigaku/MSC, 2009 ▶); cell refinement: *CrystalClear*; data reduction: *CrystalClear*; program(s) used to solve structure: *SHELXS97* (Sheldrick, 2008 ▶); program(s) used to refine structure: *SHELXL97* (Sheldrick, 2008 ▶); molecular graphics: *CrystalStructure* (Rigaku/MSC, 2009 ▶); software used to prepare material for publication: *CrystalStructure*.

## Supplementary Material

Crystal structure: contains datablock(s) I, global. DOI: 10.1107/S1600536811052299/is5001sup1.cif

Structure factors: contains datablock(s) I. DOI: 10.1107/S1600536811052299/is5001Isup2.hkl

Supplementary material file. DOI: 10.1107/S1600536811052299/is5001Isup3.cml

Additional supplementary materials:  crystallographic information; 3D view; checkCIF report

## Figures and Tables

**Table 1:** Hydrogen-bond geometry (Å, °)

*D*—H⋯*A*	*D*—H	H⋯*A*	*D*⋯*A*	*D*—H⋯*A*
N1—H1*N*⋯N3^i^	0.83 (2)	2.52 (2)	3.337 (2)	169 (2)
N2—H2*N*⋯O1^ii^	0.83 (2)	1.98 (2)	2.807 (2)	175 (2)

Symmetry codes: (i) 
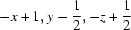
; (ii) 
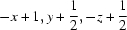
.

**Fig. 1.**
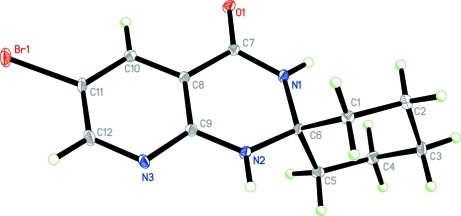
The molecular structure of the title compound with the atom numbering scheme. Displacement ellipsoids are drawn at the 30% probability level.

**Fig. 2.**
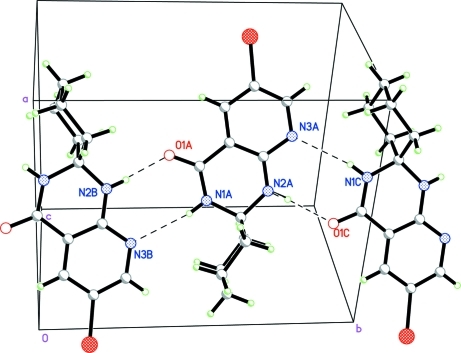
A packing diagram, showing N—H···O and N—H···N interactions (dotted lines) in the crystal structure of the title compound.

## supplementary crystallographic information

### Comment

2,3-Dihydropyrido[2,3-d]-pyrimidin-4(1*H*)-ones are a class of fused heterocycles which possess diuretic (Parish *et al.*, 1982) and anti-bacterial activity (Narayana *et al.*, 2009). 2-Substituted 2,3-dihydropyrido [2,3-d]pyrimidin-4(1*H*)-one derivatives can be obtained from the cyclocondensation of 2-amino-3-cyanopyridine with cyclopentanone (Shi *et al.*, 2010). Here, we report the crystal structure of the title compound (Fig. 1). The molecular structure is built up with two fused six-membered ring and one six-membered ring linked through a spiro C atom. The pyrimidine ring has an envelope conformation, similar to that found in spiro{cyclopentane- 1,2'(1'*H*)pyrido[2',3'-*d*]pyrimidin-4'(3'*H*)-one} (Shi *et al.*, 2010). Cyclohexane ring has a similar chair conformation as cyclohexanespiro-2'-[2',3',6',7'-tetrahydro-1'*H*- cyclopenta[*d*]pyrimidin]-4'(5'H)-one (Ling *et al.*, 2009). The crystal packing (Fig. 2) is stabilized by intermolecular N—H···O and N—H···N hydrogen bonds (Table 1).

### Experimental

A solution of 5-Br-2-amino-3-cyanopyridine (2 mmol) and sodium methylate (0.6 mmol) was refluxed in cyclohexanone (3 ml) for 10 min. The reaction mixture was cooled to room temperature and then filtered to give the title compound. The product was recrystallizated from THF to give light yellow crystalline powder (m.p. 531–532 K). ^1^H–NMR(DMSO, p.p.m.):1.30–1.73 (10*H*, m, C_5_H_10_), 7.79 (1*H*, s, NH), 7.92 (1*H*, d, J = 2.4 Hz, Pyridine-H), 8.24 (1*H*, d, J = 2.4 Hz, Pyridine-H), 8.35 (1*H*, s, NH); ESI-MS m/*z*: [*M*+H]^+^ 296.1; C_12_H_14_BrN_3_O: calcd. C 48.67, H 4.76, N 14.19; found C 48.88, H 4.787, N 14.06.

### Refinement

C-bounf H atoms were included in the riding model approximation, with C—H = 0.95–0.99 Å, and with *U*_iso_(H) = 1.2*U*_eq_(C) or 1.5*U*_eq_(methyl C), while the N-bound H atoms were refined freely [N—H = 0.83 (2) Å].

### Crystal data

**Table:**

C_12_H_14_BrN_3_O	*F*(000) = 600
*M**_r_* = 296.17	*D*_x_ = 1.665 Mg m^−^^3^
Monoclinic, *P*2_1_/*c*	Mo *K*α radiation, λ = 0.71073 Å
Hall symbol: -P 2ybc	Cell parameters from 3621 reflections
*a* = 10.591 (3) Å	θ = 2.6–29.1°
*b* = 12.359 (3) Å	µ = 3.47 mm^−^^1^
*c* = 9.116 (3) Å	*T* = 153 K
β = 97.951 (4)°	Platelet, colourless
*V* = 1181.7 (6) Å^3^	0.40 × 0.24 × 0.09 mm
*Z* = 4	

### Data collection

**Table:**

Rigaku AFC10/Saturn724+ diffractometer	3160 independent reflections
Radiation source: Rotating Anode	2283 reflections with *I* > 2σ(*I*)
graphite	*R*_int_ = 0.046
Detector resolution: 28.5714 pixels mm^-1^	θ_max_ = 29.1°, θ_min_ = 2.6°
φ and ω scans	*h* = −14→12
Absorption correction: multi-scan (*CrystalClear*; Rigaku/MSC, 2009)	*k* = −16→16
*T*_min_ = 0.324, *T*_max_ = 0.732	*l* = −12→12
10128 measured reflections	

### Refinement

**Table:**

Refinement on *F*^2^	Primary atom site location: structure-invariant direct methods
Least-squares matrix: full	Secondary atom site location: difference Fourier map
*R*[*F*^2^ > 2σ(*F*^2^)] = 0.035	Hydrogen site location: inferred from neighbouring sites
*wR*(*F*^2^) = 0.074	H atoms treated by a mixture of independent and constrained refinement
*S* = 1.00	*w* = 1/[σ^2^(*F*_o_^2^) + (0.0295*P*)^2^ + 0.126*P*] where *P* = (*F*_o_^2^ + 2*F*_c_^2^)/3
3160 reflections	(Δ/σ)_max_ = 0.001
162 parameters	Δρ_max_ = 0.55 e Å^−^^3^
0 restraints	Δρ_min_ = −0.66 e Å^−^^3^

### Special details

**Table:**

Experimental. Spectral data: IR (KBr): 3274, 3175, 2927, 1677, 1610, 1422 cm^-1^.
Geometry. All e.s.d.'s (except the e.s.d. in the dihedral angle between two l.s. planes) are estimated using the full covariance matrix. The cell e.s.d.'s are taken into account individually in the estimation of e.s.d.'s in distances, angles and torsion angles; correlations between e.s.d.'s in cell parameters are only used when they are defined by crystal symmetry. An approximate (isotropic) treatment of cell e.s.d.'s is used for estimating e.s.d.'s involving l.s. planes.
Refinement. Refinement of *F*^2^ against ALL reflections. The weighted *R*-factor *wR* and goodness of fit *S* are based on *F*^2^, conventional *R*-factors *R* are based on *F*, with *F* set to zero for negative *F*^2^. The threshold expression of *F*^2^ > σ(*F*^2^) is used only for calculating *R*-factors(gt) *etc*. and is not relevant to the choice of reflections for refinement. *R*-factors based on *F*^2^ are statistically about twice as large as those based on *F*, and *R*- factors based on ALL data will be even larger.

### Fractional atomic coordinates and isotropic or equivalent isotropic displacement parameters (Å^2^)

**Table:**

	*x*	*y*	*z*	*U*_iso_*/*U*_eq_	
Br1	0.99820 (2)	0.65490 (2)	0.61599 (3)	0.03640 (10)	
O1	0.58435 (14)	0.40734 (11)	0.39085 (16)	0.0171 (3)	
N1	0.45035 (17)	0.52063 (14)	0.2500 (2)	0.0151 (4)	
N2	0.49356 (17)	0.71034 (14)	0.2458 (2)	0.0188 (4)	
N3	0.68151 (16)	0.78374 (13)	0.3597 (2)	0.0176 (4)	
C1	0.2735 (2)	0.64979 (16)	0.2411 (2)	0.0150 (4)	
H1A	0.2814	0.6418	0.3501	0.018*	
H1B	0.2496	0.7257	0.2164	0.018*	
C2	0.1676 (2)	0.57477 (17)	0.1687 (2)	0.0179 (5)	
H2A	0.1865	0.4994	0.2015	0.021*	
H2B	0.0855	0.5960	0.2005	0.021*	
C3	0.1565 (2)	0.58081 (18)	−0.0002 (2)	0.0194 (5)	
H3A	0.0902	0.5296	−0.0448	0.023*	
H3B	0.1303	0.6547	−0.0336	0.023*	
C4	0.2831 (2)	0.55308 (17)	−0.0524 (2)	0.0172 (5)	
H4A	0.2751	0.5617	−0.1613	0.021*	
H4B	0.3046	0.4765	−0.0285	0.021*	
C5	0.3902 (2)	0.62565 (16)	0.0209 (2)	0.0160 (4)	
H5A	0.3743	0.7006	−0.0152	0.019*	
H5B	0.4717	0.6014	−0.0098	0.019*	
C6	0.40340 (19)	0.62564 (15)	0.1905 (2)	0.0129 (4)	
C7	0.55747 (19)	0.49967 (15)	0.3433 (2)	0.0127 (4)	
C8	0.64262 (19)	0.59204 (16)	0.3832 (2)	0.0125 (4)	
C9	0.6063 (2)	0.69577 (16)	0.3293 (2)	0.0139 (4)	
C10	0.7594 (2)	0.57798 (17)	0.4683 (2)	0.0174 (5)	
H10	0.7863	0.5086	0.5051	0.021*	
C11	0.8365 (2)	0.66793 (17)	0.4987 (3)	0.0203 (5)	
C12	0.7942 (2)	0.76778 (17)	0.4435 (3)	0.0203 (5)	
H12	0.8482	0.8286	0.4665	0.024*	
H1N	0.408 (2)	0.4668 (17)	0.220 (2)	0.015 (6)*	
H2N	0.474 (2)	0.7700 (19)	0.208 (3)	0.026 (7)*	

### Atomic displacement parameters (Å^2^)

**Table:**

	*U*^11^	*U*^22^	*U*^33^	*U*^12^	*U*^13^	*U*^23^
Br1	0.02057 (13)	0.02781 (14)	0.05373 (19)	−0.00860 (11)	−0.01997 (11)	0.01191 (13)
O1	0.0199 (8)	0.0096 (7)	0.0194 (8)	0.0000 (6)	−0.0054 (6)	0.0021 (6)
N1	0.0143 (9)	0.0080 (8)	0.0209 (10)	−0.0027 (7)	−0.0049 (7)	−0.0002 (7)
N2	0.0163 (10)	0.0070 (9)	0.0298 (11)	−0.0006 (7)	−0.0085 (8)	0.0047 (8)
N3	0.0143 (10)	0.0111 (9)	0.0259 (11)	−0.0045 (7)	−0.0027 (8)	0.0016 (8)
C1	0.0165 (11)	0.0128 (10)	0.0153 (10)	0.0017 (9)	0.0011 (8)	−0.0012 (8)
C2	0.0133 (11)	0.0189 (11)	0.0216 (12)	−0.0025 (9)	0.0031 (9)	−0.0022 (9)
C3	0.0130 (11)	0.0208 (11)	0.0227 (12)	−0.0036 (9)	−0.0029 (9)	−0.0027 (10)
C4	0.0194 (11)	0.0184 (10)	0.0131 (10)	0.0000 (9)	−0.0003 (8)	−0.0017 (9)
C5	0.0167 (11)	0.0145 (10)	0.0175 (11)	0.0032 (8)	0.0050 (9)	0.0026 (9)
C6	0.0117 (10)	0.0070 (9)	0.0182 (11)	0.0004 (8)	−0.0042 (8)	0.0004 (8)
C7	0.0135 (10)	0.0112 (10)	0.0132 (10)	0.0009 (8)	0.0013 (8)	−0.0005 (8)
C8	0.0129 (10)	0.0113 (10)	0.0130 (10)	−0.0002 (8)	0.0006 (8)	−0.0005 (8)
C9	0.0137 (10)	0.0111 (9)	0.0165 (11)	−0.0006 (8)	0.0010 (8)	−0.0003 (8)
C10	0.0153 (11)	0.0151 (10)	0.0204 (11)	−0.0008 (9)	−0.0020 (9)	0.0041 (9)
C11	0.0120 (10)	0.0200 (11)	0.0262 (12)	−0.0040 (9)	−0.0064 (9)	0.0027 (10)
C12	0.0161 (11)	0.0148 (10)	0.0281 (13)	−0.0052 (9)	−0.0034 (9)	−0.0019 (10)

### Geometric parameters (Å, °)

**Table:**

Br1—C11	1.896 (2)	C3—C4	1.523 (3)
O1—C7	1.240 (2)	C3—H3A	0.9900
N1—C7	1.346 (2)	C3—H3B	0.9900
N1—C6	1.467 (2)	C4—C5	1.525 (3)
N1—H1N	0.83 (2)	C4—H4A	0.9900
N2—C9	1.336 (3)	C4—H4B	0.9900
N2—C6	1.459 (3)	C5—C6	1.533 (3)
N2—H2N	0.83 (2)	C5—H5A	0.9900
N3—C12	1.339 (3)	C5—H5B	0.9900
N3—C9	1.354 (3)	C7—C8	1.469 (3)
C1—C2	1.533 (3)	C8—C10	1.377 (3)
C1—C6	1.539 (3)	C8—C9	1.407 (3)
C1—H1A	0.9900	C10—C11	1.384 (3)
C1—H1B	0.9900	C10—H10	0.9500
C2—C3	1.530 (3)	C11—C12	1.384 (3)
C2—H2A	0.9900	C12—H12	0.9500
C2—H2B	0.9900		
			
C7—N1—C6	128.11 (17)	C4—C5—C6	113.60 (18)
C7—N1—H1N	115.1 (15)	C4—C5—H5A	108.8
C6—N1—H1N	116.7 (15)	C6—C5—H5A	108.8
C9—N2—C6	126.21 (17)	C4—C5—H5B	108.8
C9—N2—H2N	120.3 (17)	C6—C5—H5B	108.8
C6—N2—H2N	112.6 (17)	H5A—C5—H5B	107.7
C12—N3—C9	116.82 (17)	N2—C6—N1	109.55 (16)
C2—C1—C6	112.62 (16)	N2—C6—C5	108.24 (17)
C2—C1—H1A	109.1	N1—C6—C5	110.62 (17)
C6—C1—H1A	109.1	N2—C6—C1	109.04 (16)
C2—C1—H1B	109.1	N1—C6—C1	109.38 (17)
C6—C1—H1B	109.1	C5—C6—C1	109.98 (16)
H1A—C1—H1B	107.8	O1—C7—N1	122.07 (18)
C3—C2—C1	110.73 (17)	O1—C7—C8	121.69 (18)
C3—C2—H2A	109.5	N1—C7—C8	116.22 (17)
C1—C2—H2A	109.5	C10—C8—C9	119.49 (18)
C3—C2—H2B	109.5	C10—C8—C7	120.94 (18)
C1—C2—H2B	109.5	C9—C8—C7	119.53 (18)
H2A—C2—H2B	108.1	N2—C9—N3	117.56 (18)
C4—C3—C2	110.80 (17)	N2—C9—C8	120.06 (18)
C4—C3—H3A	109.5	N3—C9—C8	122.37 (18)
C2—C3—H3A	109.5	C8—C10—C11	118.05 (19)
C4—C3—H3B	109.5	C8—C10—H10	121.0
C2—C3—H3B	109.5	C11—C10—H10	121.0
H3A—C3—H3B	108.1	C12—C11—C10	119.4 (2)
C3—C4—C5	111.35 (17)	C12—C11—Br1	120.17 (16)
C3—C4—H4A	109.4	C10—C11—Br1	120.42 (16)
C5—C4—H4A	109.4	N3—C12—C11	123.84 (19)
C3—C4—H4B	109.4	N3—C12—H12	118.1
C5—C4—H4B	109.4	C11—C12—H12	118.1
H4A—C4—H4B	108.0		
			
C6—C1—C2—C3	−56.1 (2)	N1—C7—C8—C10	−174.77 (19)
C1—C2—C3—C4	56.8 (2)	O1—C7—C8—C9	−178.41 (19)
C2—C3—C4—C5	−55.7 (2)	N1—C7—C8—C9	3.0 (3)
C3—C4—C5—C6	54.2 (2)	C6—N2—C9—N3	174.7 (2)
C9—N2—C6—N1	4.4 (3)	C6—N2—C9—C8	−5.9 (3)
C9—N2—C6—C5	−116.3 (2)	C12—N3—C9—N2	180.0 (2)
C9—N2—C6—C1	124.1 (2)	C12—N3—C9—C8	0.6 (3)
C7—N1—C6—N2	1.1 (3)	C10—C8—C9—N2	179.6 (2)
C7—N1—C6—C5	120.3 (2)	C7—C8—C9—N2	1.8 (3)
C7—N1—C6—C1	−118.4 (2)	C10—C8—C9—N3	−1.0 (3)
C4—C5—C6—N2	−170.86 (16)	C7—C8—C9—N3	−178.8 (2)
C4—C5—C6—N1	69.1 (2)	C9—C8—C10—C11	0.6 (3)
C4—C5—C6—C1	−51.8 (2)	C7—C8—C10—C11	178.3 (2)
C2—C1—C6—N2	171.29 (17)	C8—C10—C11—C12	0.2 (3)
C2—C1—C6—N1	−68.9 (2)	C8—C10—C11—Br1	179.67 (16)
C2—C1—C6—C5	52.7 (2)	C9—N3—C12—C11	0.3 (3)
C6—N1—C7—O1	176.9 (2)	C10—C11—C12—N3	−0.6 (4)
C6—N1—C7—C8	−4.5 (3)	Br1—C11—C12—N3	179.88 (18)
O1—C7—C8—C10	3.8 (3)		

### Hydrogen-bond geometry (Å, °)

**Table:**

*D*—H···*A*	*D*—H	H···*A*	*D*···*A*	*D*—H···*A*
N1—H1N···N3^i^	0.83 (2)	2.52 (2)	3.337 (2)	169 (2)
N2—H2N···O1^ii^	0.83 (2)	1.98 (2)	2.807 (2)	175 (2)

Symmetry codes: (i) −*x*+1, *y*−1/2, −*z*+1/2; (ii) −*x*+1, *y*+1/2, −*z*+1/2.
